# The comparison of labor work training performances for the patients with mental illness in a public psychiatric center

**DOI:** 10.1192/j.eurpsy.2025.2170

**Published:** 2025-08-26

**Authors:** F.-Y. Gu, A.-L. Liu, W.-C. Lue

**Affiliations:** 1 Occupational Therapy, Taipei City Hospital, Songde Branch; 2 Taipei City Hospital Songde Branch Psychiatric Nursing Home, Taipei City, Taiwan, Province of China

## Abstract

**Introduction:**

Mental illness is a kind of change in mental health status which affected thought, feeling, emotion, behaviors, and so on. Labor work training is one of common rehabilitation programs in psychiatric medical institutes in Taiwan. This rehabilitation program has been implemented for many years, being an important indicator of psychiatric hospital accreditation. However, we couldn’t analysis and apply effectively.

**Objectives:**

We aim to investigate different labor work training performances of the patients with mental illness in a public psychiatric center in Taipei City. Furthermore, we can apply in shared decision making and modifying the labor work training project according to the data results.

**Methods:**

In this cross-sectional study, 89 participants with mental illness who were stably engaged in labor work training for at least 1 month were included in Taipei City Hospital Songde Branch. Descriptive statistics will be used in characteristic of population, incentive record and score of the comprehensive occupational therapy evaluation scale (COTES). We used one-way ANOVA to test the difference of work behavior scores in different labor work training.

**Results:**

This study results showed that the percentage of participants in the cleaning labor group, manual group, and sales & delivery group were 15% (N=13), 49% (N=44), and 36% (N=32) respectively. The most people engaged in the manual group. The average total score of psychiatric patients’ work behavior was highest in the sales & delivery group (*p*<.05). There were significant differences at scores of work behavior items, including motivation (*p*=.006)、duration (*p*=.001), responsibility (*p*=.002), comprehension (*p*=.002), technique (*p*=.005), and fine motor coordination (*p*=.005).

**Image 1:**

**Figure:**
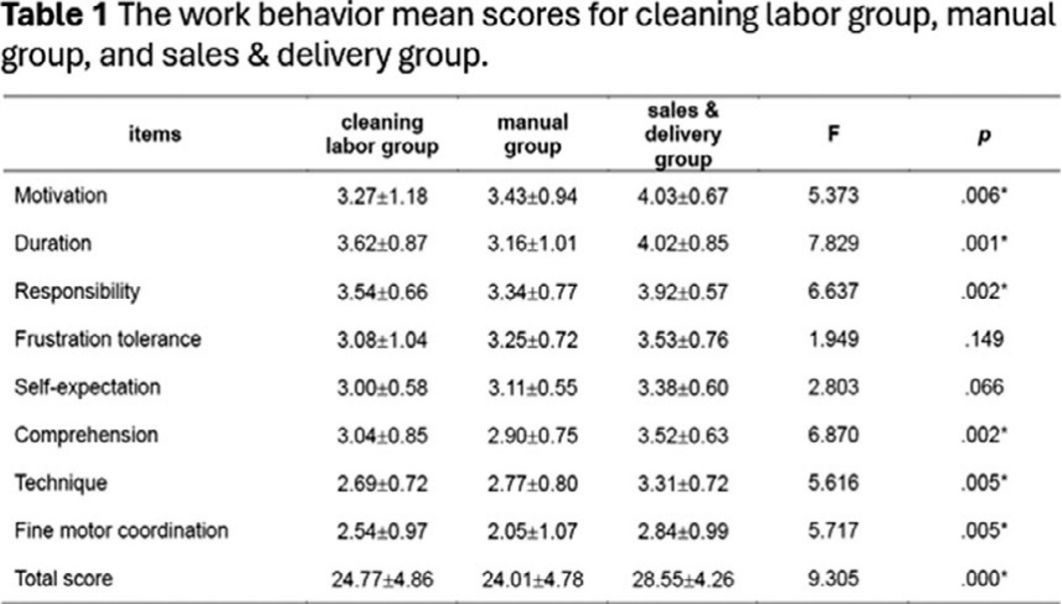

**Image 2:**

**Figure:**
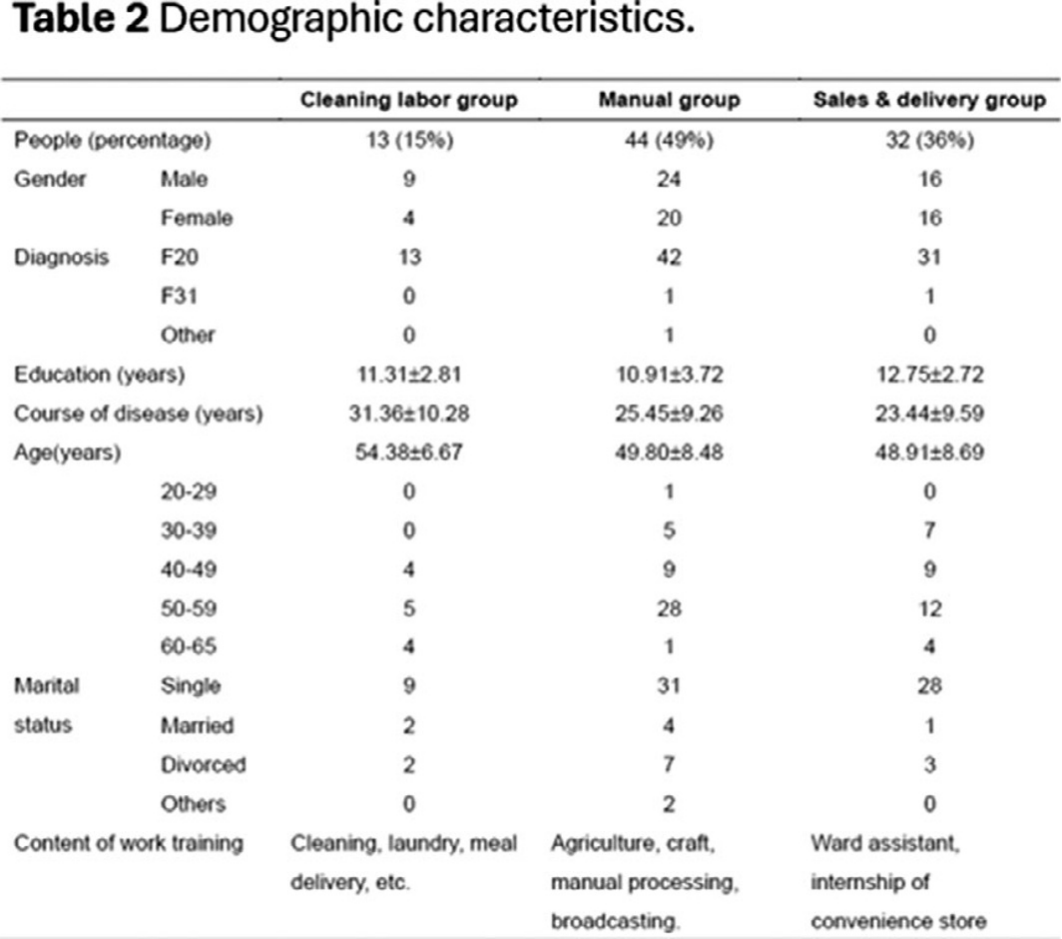

**Conclusions:**

In comparison, the patients with mental illness in the sales & delivery group are better at work behavior. According to the analysis of the result data, when the therapist and the patient make shared decisions, they can discuss appropriate labor work training based on the patient’s work behavior.

**Disclosure of Interest:**

None Declared

